# Markedly Increased IP-10 Production by Blood-Brain Barrier in Neuromyelitis Optica

**DOI:** 10.1371/journal.pone.0122000

**Published:** 2015-03-26

**Authors:** Fumitaka Shimizu, Hideaki Nishihara, Yasuteru Sano, Yukio Takeshita, Shiori Takahashi, Toshihiko Maeda, Toshiyuki Takahashi, Masaaki Abe, Michiaki Koga, Takashi Kanda

**Affiliations:** 1 Department of Neurology and Clinical Neuroscience, Yamaguchi University Graduate School of Medicine, Ube, Japan; 2 Department of Neurology, Tohoku University Graduate School of Medicine, Miyagi, Japan; Medical University Vienna, Center for Brain Research, AUSTRIA

## Abstract

**Objective:**

Severe damage to the blood-brain barrier (BBB) allows anti-aquaporin 4 (AQP4) antibodies to access the astrocytic endfeet in neuromyelitis optica (NMO). In the current study, we identified the pathogenic cytokines/chemokines that are responsible for the BBB malfunction induced by NMO sera.

**Methods:**

We measured the levels of 27 cytokines/chemokines in human brain microvascular endothelial cells (BMECs) after exposure to sera obtained from patients with the acute and stable phases of anti-AQP4 antibody-positive NMO spectrum disorder (NMOSD), multiple sclerosis (MS) patients and healthy controls (HC) using a multiplexed fluorescent bead-based immunoassay system.

**Results:**

The induced protein (IP)-10 level in the cells was markedly increased following exposure to acute phase NMOSD sera. Other cytokines/chemokines including interleukin (IL)-6 and monocyte chemotactic protein (MCP)-1 were also significantly increased in the acute NMOSD group compared to both the MS and HC groups. The up-regulation of the IP-10 levels in the cells after exposure to the acute-phase NMOSD sera was also observed using another specified ELISA, and this effect was significantly decreased during the remission phase in the individual NMOSD patients. Furthermore, the increase in the level of IP-10 after exposure to the sera was significantly correlated with the cerebrospinal fluid/serum albumin ratio.

**Conclusions:**

Sera from the acute phase of NMO markedly increased the autocrine secretion of IP-10 by BMECs. The over-production of IP-10 in BMECs may play an important role in the pathogenesis of NMO and may therefore help to mediate the trafficking of T cells expressing its receptor across the BBB.

## Introduction

Neuromyelitis optica (NMO) is an inflammatory disorder of the central nervous system (CNS) that preferentially affects the optic nerves and spinal cord, leading to a loss of visual and motor function [1,2]. The discovery of novel and disease-specific serum anti-aquaporin (AQP) 4 antibodies has clearly identified NMO as a separate disease entity from MS, and suggested that AQP4 is a specific immunological target in NMO [3]. A pathogenic role of anti-AQP4 antibodies in the development of NMO has been demonstrated both *in vitro*, by the fact that caused complement-mediated astrocyte cytotoxicity [4–6], and *in vivo*, by passive transfer experiments in animal models [7–9]. However, undetermined factors other than anti-AQP4 antibodies including inflammatory mediators, T and B cell involvement and blood-brain barrier (BBB) disruption, is required to trigger the development of the disease, because the presence of serum anti-AQP4 antibodies alone is insufficient to cause NMO without inflammation [10–12].

Many studies have demonstrated that there are increased levels of some cytokines and chemokines in the cerebrospinal fluid (CSF) of NMO patients, and these studies have focused on the additional inflammatory and pathological biomarkers of NMO [13–17]. For example, the CSF interleukin (IL)-6 levels in NMO patients were significantly higher compared to those in patients with MS or other non-inflammatory neurological disorders, and were significantly correlated with clinical variables, including the Expanded Disability Status Scale (EDSS) score, CSF glial fibrillary acidic protein (GFAP) level and anti-AQP4 antibody titers [15–17]. These data are practically useful for understanding the pathogenic and immunological aspects of NMO, but have limitations, because the causative role of CSF cytokines in NMO patients is unclear, and while they may be increased as important pathogenic molecules, it is also possible that they are merely a byproduct of inflammation.

The destruction of the BBB, which allows the penetration of circulating anti-AQP4 antibodies into the CNS space, is thought to be associated with the pathogenesis of NMO [18,19]. Our previous studies demonstrated that sera from NMO spectrum disorder (NMOSD) patients induces BBB malfunction via the autocrine secretion of vascular endothelial growth factors (VEGF) and matrix metalloproteinase-2/9 by the microvascular endothelial cells of the brain (BMECs) [20,21], suggesting that a focal increase of the cytokines/chemokines around the BBB may be involved in the pathogenesis of NMO. In the present study, we measured the production of cytokines/chemokines secreted by BMECs after exposure to sera obtained from patients with NMO, MS and healthy controls.

## Materials and Methods

### Sera

This study was approved by the ethics committee, of the Medical Faculty, Yamaguchi University, and written informed consent was obtained from each participant. Sera were collected from 20 NMOSD patients diagnosed at Yamaguchi University Hospital based on the revised criteria for NMOSD [1] and who exhibited seropositivity for anti-AQP4 antibodies using an immunofluorescence method, as described previously (three males, 17 females; mean age, 53.2 years) [22]. The 20 samples collected during the acute phase were obtained within one month of the initiation of attack, and included 11 samples from patients with definite NMO, five samples from those with isolated longitudinally extensive transverse myelitis (LETM) and four samples from those with isolated optic neuritis (ON). Eight longitudinal samples were obtained from NMOSD patients in the remission phase, who were being treated with a corticosteroid after relapse and had been in clinical remission for at least six months. Cerebrospinal fluid (CSF) samples were concurrently obtained from 18 NMOSD patients within one month of the onset of attacks. We recorded the disease duration (mean disease duration, 3.9 year (SD = ±5.6)), number of relapses (median number of relapses, 3.0 times (SD = ±3.1)), change in the score on the Expanded Disability Status Scale (EDSS) from before to after relapse (ΔEDSS) (mean ΔEDSS, 5.2 (SD = ±2.9)), IgG index as the marker of intrathecal IgG synthesis (median IgG index, 0.57 (SD = ±0.21)), CSF/serum albumin ratio (Q Alb) as a marker of BBB integrity (median Q Alb, 0.007 (SD = ±0.003)), length of the spinal cord lesion on MRI (median vertebral segment, 3.95 (SD = ±3.73)) and presence of Gd-enhanced lesions on MRI in the 20 NMOSD patients. Serum samples were also collected within one week after relapse from six patients with acute phase of relapse-remiting (RR) MS, all of whom fulfilled the revised McDonald criteria [23] and presented with both new worsening of neurological symptoms associated with objective neurological signs and the appearance of a new Gd-enhancing lesion on MRI (three males, 3 females; mean age, 37.8 years; mean disease duration, 4.5 year (SD = ±4.6); median number of relapses, 3.0 times (SD = ±2.1)). In addition, 10 individuals served as healthy controls (HCs) (four males, 6 females; mean ages, 32.6 year). All samples were immediately stored at -80°C until the analysis and were inactivated at 56°C for 30 minutes immediately before the analysis.

### Cell culture and treatment

Conditionally immortalized human BMECs, named “TY09 cells”, which retain the temperature-sensitive SV40 large T antigen (tsA58) protein, have been described previously [24]. Briefly, these cells show immortality at the permissive temperature of 33°C, as the gene product of tsA58 is on an active conformation and binds to p53 at 33°C. However, the conformation of the tsA58 gene product was degraded and p53 was released when the cells were grown at 37°C, suggesting that these cell lines maintain the physiological properties of the normal brain endothelial cells at 37°C. In addition, the cell lines had key tight junction proteins including claudin-5, occludin, ZO-1 and ZO-2 and transporters including GLUT-1 and p-glycoprotein and showed low permeability to inulin across the monolayer [24]. All analyses were performed three days after the temperature shift from 33°C to 37°C. The cells were cultured in conditioned medium containing 10% serum obtained from the patients with NMO during the acute and stable phases, those with RRMS in the acute phase or the healthy controls in a CO_2_ incubator at 37°C for 24 hours. The addition of sera did not seem to have any functional effect on the cell morphology. The conditioned media were completely removed and washed with PBS solution several times, and the total proteins were extracted from the cells one day later.

### Multiplexed fluorescent bead-based immunoassay

The concentrations of 27 cytokines/chemokines in equal amounts of protein (22.5 μg) from each sample obtained from the TY09 cells after exposure to sera were measured using the Bio-Plex human 27-Plex cytokine panels and a Bio-Plex cytokine reagent kit (Bio-Rad, Hercules, CA) according to the manufacturer’s instructions. The levels of interleukin (IL)-1β, IL-2, IL-4, IL-5, IL-6, IL-7, CXCL8/IL-8, IL-9, IL-10, IL-12 (p70), IL-13, IL-15, IL-17, IL-1 receptor antagonist (IL-1ra), tumor necrosis factor (TNF)-α, interferon (IFN)-γ, fibroblast growth factor (FGF)-2, VEGF, CCL11/eotaxin, granulocyte colony-stimulating factor (G-CSF), granulocyte-macrophage colony-stimulating factor (GM-CSF), platelet-derived growth factor (PDGF)-11, CXCL10/IFN-inducible protein of 10 kDa (IP-10), CCL2/macrophage chemoattractant protein-1 (MCP-1), CCL3/macrophage inflammatory protein (MIP)-1α, CCL4/MIP-1β and CCL5/regulated upon activation, normal T cell expressed and secreted (RANTES) were analyzed in this study. The concentrations of cytokines/chemokines were calculated by referencing a standard curve for each set of molecules derived from various concentrations of the standard assays.

### ELISA anaylysis

The serum concentration of IP-10 and concentration of equal amounts of protein (22.5 μg) from each sample obtained from the TY09 cells after exposure to sera were measured by an ELISA using commercially available kits (R&D Systems, Minneapolis, Minnesota, USA). The results were expressed as picograms of IP-10 per milliliter (pg/ml), based on reference to the standard curves provided with the available kits. All samples were analyzed in duplicate.

### Data analysis

All comparisons of the median values between the groups were analyzed by the Mann-Whitney U test for continuous variables, and a two-sided value of p<0.05 was considered to be statistically significant. Pearson correlation coefficients were used to test the associations between the clinical, laboratory and MRI findings and the level of IP-10 produced by the cells.

## Results

### Comparison of the cytokine/chemokine profile in protein samples from BMECs after exposure to sera from patients with acute and stable NMOSD, RRMS and healthy controls

We measured the concentrations of 27 cytokines/chemokines in protein samples obtained from the TY09 cells after exposure to sera from NMOSD patients obtained during the acute phase (acute NMOSD group), from NMOSD patients obtained during the stable phase (stable NMOSD group), RRMS patients (RRMS group) and healthy volunteers (HC group). Among the 27 cytokines, IL-15, IL-17, GM-CSF and MIP-1b were not detected in this assay (S1 **Dataset**). Table 1 demonstrates the profiles of the other 23 cytokines/chemokines detected in the TY09 cells. A comparison of the levels of 14 cytokines/chemokines did not show any statistically significant differences between the groups.

**Table 1:** Summary of cytokine and chemokine levels secreted by BMECs after exposure to patient’s sera.

	HC (n = 10)	RRMS (n = 6)	P value (vs HC)	Acute NMO (n = 20)	P value (vs HC)	Stable NMO (n = 8)	P value (vs HC)
	Mean (SD)	Mean (SD)		Mean (SD)		Mean (SD)	
Increased in acute NMO compared to HC							
IP-10	117 (20)	108 (10)	NS	402 (151)	<0.0001	108 (94)	NS
Increased in acute NMO and decreased in stable NMO compared to HC							
IL-6	49.5 (12.2)	52.2 (14.0)	NS	83.5	<0.001	30.0	<0.01
MCP-1	20.7 (6.2)	21.7 (7.0)	NS	37.0 (14.6)	<0.001	9.0 (8.0)	<0.01
Increased in both acute NMO and stable NMO compared to HC							
RANTES	201 (43)	209 (84)	NS	438 (152)	<0.0001	341 (163)	<0.01
Increased in acute NMO, MS and stable NMO compared to HC							
PDGF-BB	33.9 (8.9)	59.9 (24.5)	<0.05	44.0 (8.4)	<0.01	51.0 (18.0)	<0.05
Increased in both MS and stable NMO compared to HC							
VEGF	230 (22)	301 (61)	<0.01	251 (38)	NS	347 (39)	<0.001
Decreased in acute NMO compared to HC							
IL-1β	1.74 (0.30)	1.72 (0.26)	NS	1.34 (1.43)	<0.01	1.90 (0.23)	NS
IL-10	61.9 (20.8)	62.1 (11.0)	NS	36.4 (18.8)	<0.01	68.8 (22.2)	NS
TNF-α	705 (140)	617 (81)	NS	550 (128)	<0.05	643 (169)	NS
Decreased in both acute NMO and MS compared to HC							
IL-4	2.83 (1.76)	1.12 (1.24)	<0.05	0.70 (1.42)	<0.01	2.30 (2.50)	NS
Unvaried between acute NMO and MS, stable NMO or HC							
IL-5	1.55 (0.87)	1.40 (0.17)	NS	1.38 (0.54)	NS	1.47 (1.05)	NS
IL-7	21.4 (2.2)	20.4 (4.0)	NS	20.8 (2.7)	NS	20.8 (2.7)	NS
IL-8	35.0 (6.0)	31.5 (12.4)	NS	36.4 (17.7)	NS	26.6 (9.5)	NS
IL-9	5.98 (2.12)	7.23 (1.73)	NS	6.04 (2.05)	NS	4.72 (3.08)	NS
IL-12	21.5 (5.5)	24.2 (3.9)	NS	20.3 (4.8)	NS	27.0 (6.0)	<0.05
IL-13	3.00 (0.56)	2.76 (0.40)	NS	2.55 (0.38)	NS	2.82 (0.82)	NS
IL-1ra	117 (69)	126 (18)	NS	93 (39)	NS	126 (18)	NS
FGF-2	493 (117)	619 (82)	NS	447 (68)	NS	354 (85)	NS
Eotaxin	12.4 (5.0)	11.6 (2.6)	NS	8.81 (3.93)	NS	11.6 (5.8)	NS
G-CSF	548 (103)	478 (54)	NS	545 (66)	NS	466 (106)	NS
MIP-1a	2.90 (1.35)	3.13 (0.46)	NS	2.31 (1.26)	NS	2.55 (1.94)	NS
MIP-1b	0.23 (0.50)	0 (0)	NS	0 (0)	NS	0.23 (0.44)	NS
Undetermined cytokines: IL-15, IL-17, GM-CSF and MIP-1b							

Abbreviations: HC = healthy control; RRMS = relapse-remiting multiple sclerosis; acute NMO = neuromyelitis optica in the acute phase; stable NMO = neuromyelitis optica in the stable phase; NS = not significant; IP = induced protein; IL = interleukin; MCP = monocyte chemotactic protein; RANTES = regulated upon activation, normal T cell expressed and secreted; PDGF = platelet-derived growth factor; VEGF = vascular endothelial growth factor; TNF = tumor necrosis factor; IL-1ra = interleukin-1 receptor antagonist; FGF = fibroblast growth factor; G-CSF = granulocyte colony-stimulating factor; MIP = macrophage inflammatory protein; GM-CSF = granulocyte-macrophage colony-stimulating factor Unit: pg/ml

**Fig 1:**
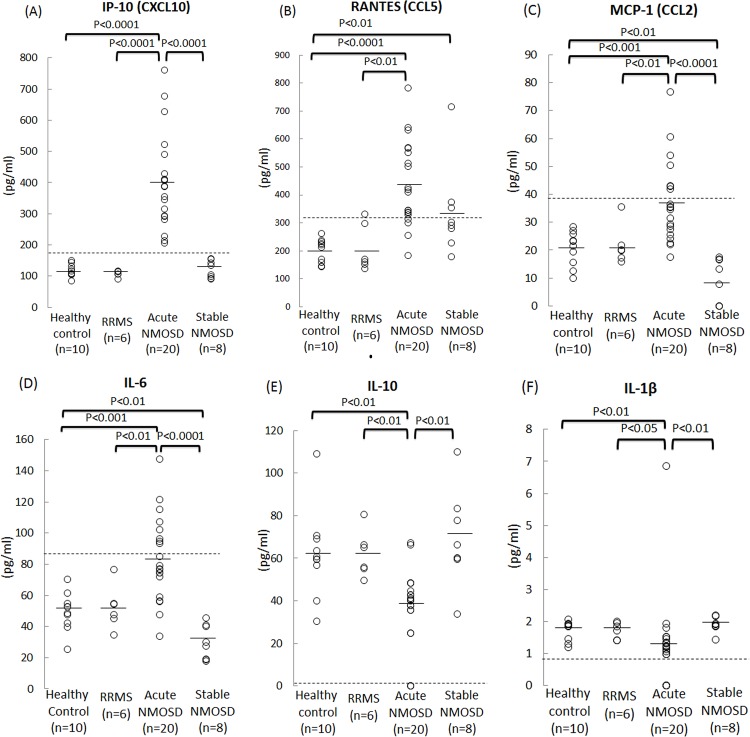
Cytokine/chemokine levels in protein samples from BMECs after exposure to sera from patients with acute and stable NMOSD, RRMS and healthy controls. The concentrations of cytokines/chemokines including IP-10 (A), RANTES (B), MCP-1 (C), IL-6 (D), IL-1β (E) and IL-10 (F) in protein samples obtained from BMECs after exposure to sera from NMOSD patients during the acute phase (acute NMOSD group), NMOSD patients during the stable phase (stable NMOSD group), relapse-remiting (RR) MS patients (RRMS group) and healthy volunteers (HC group). These six cytokines/chemokines were significantly increased/decreased in the acute NMOSD group compared to both the RRMS and HC groups. Of these six cytokines/chemokines, the value of IP-10 produced by BMECs was markedly increased in the cells treated with all of the samples from the acute NMOSD group. The dashed lines indicate the upper or lower limits of the cytokine levels in the HC group (mean±3SD). Healthy control: cells exposed to sera from healthy controls; RRMS: cells exposed to sera from patients with RRMS; Acute NMO: cells exposed to sera from NMOSD patients during the acute phase; Stable NMO: cells exposed to sera from NMOSD patients in the stable phase.

The protein levels of IL-6, MCP-1, RANTES, PDGF-BB and IP-10 produced by TY09 cells in the acute NMOSD group were significantly increased compared to those in the HC group (**Table 1**). In contrast, the protein levels of IL-1β, IL-4, IL-10 and TNF-α in the cells in the acute NMOSD group were significantly lower than those in the HC group (**Table 1**). The VEGF protein level in both the RRMS group and stable NMOSD group was significantly higher compared to that in the HC group (**Table 1**). Fig. 1 shows that six of the cytokines/chemokines were significantly increased or decreased in the acute NMOSD group compared to both the RRMS and HC groups (**Fig. 1A-F**). Out of these six cytokines/chemokines, the amount of IP-10 produced by BMECs (range, 206–761 pg/mL) was markedly increased in all of the samples in the acute NMOSD group (**Fig. 1A**), and were all above the upper normal limits of the HC group (144 pg/mL), RRMS group (117 pg/mL) and stable NMOSD group (155 pg/mL).

The values of three cytokines/chemokines including RANTES (range, 185–641 pg/mL), MCP-1 (range, 17.6–76.7 pg/mL) and IL-6 (range, 34.0–115 pg/mL), in the acute NMOSD group were also significantly increased (**Fig. 1B-D**), while the levels of IL-1β (range, 0–6.86 pg/mL) and IL-10 (range, 0–67.1 pg/mL) in these groups were significantly decreased as a group (**Fig. 1E,F**), although the levels of these cytokines remained within the normal ranges in some samples in the acute NMOSD group (the upper limits: RANTES, 261 pg/mL; MCP-1, 28.4 pg/mL; IL-6, 70.1 pg/mL; the lower limits: IL-1β, 1.30 pg/mL; IL-10, 30.3 pg/mL).

**Fig 2:**
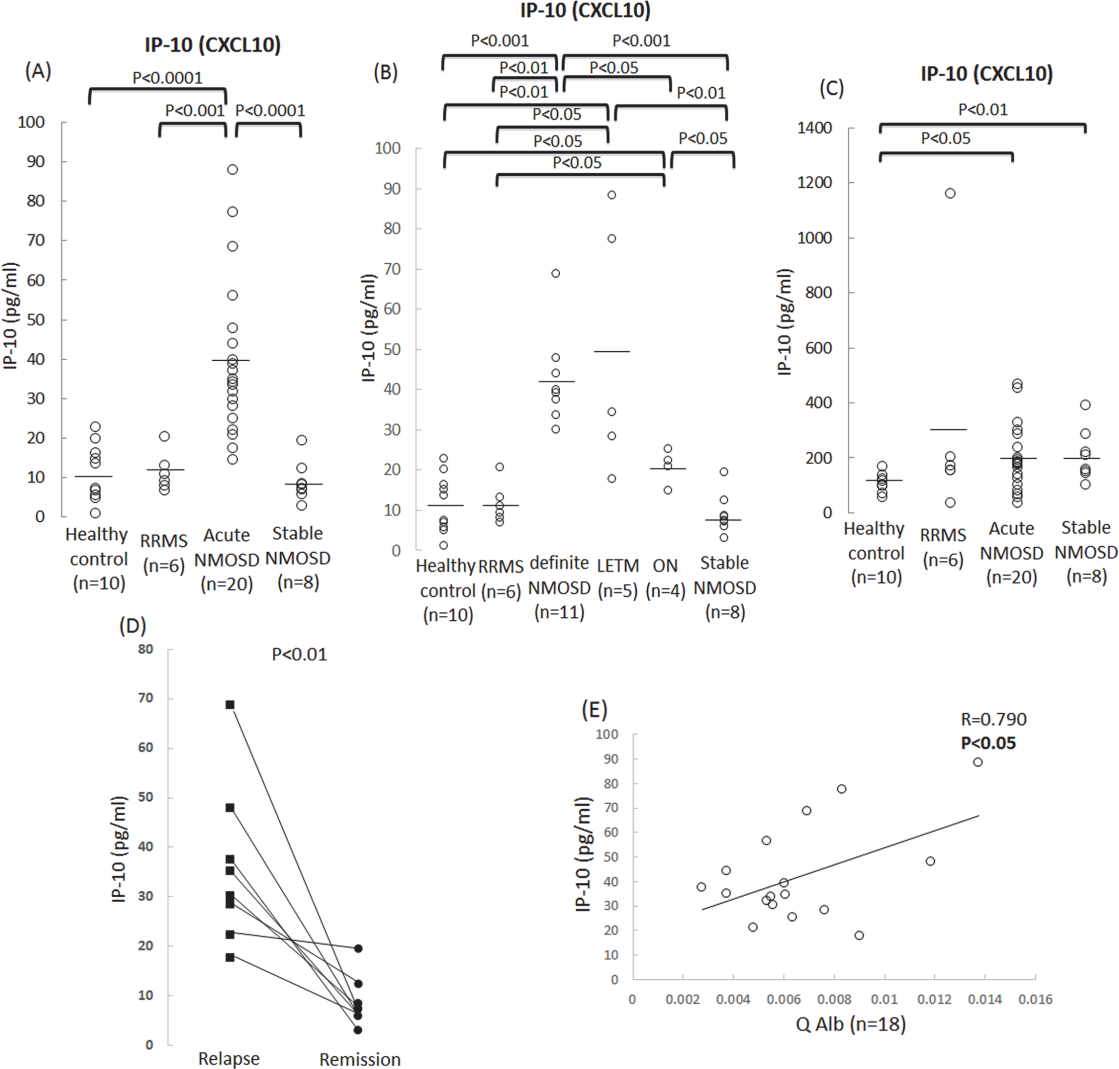
Association between the concentration of IP-10 produced by BMECs after NMOSD sera and clinical phenotypes/clinical courses/CSF parameter in NMOSD patients. (A) The IP-10 protein levels produced by the cells in the acute NMOSD group were significantly increased compared to that observed in the RRMS, stable NMO and HC groups using another specified ELISA assay. (B) The IP-10 protein levels in the cells in the definite NMOSD, isolated longitudinally extensive transverse myelitis (LETM) and optic neuritis (ON) group were significantly increased compared to that seen in the RRMS and HC groups. The increase in the protein levels of IP-10 was significantly greater after incubation with definite NMOSD than that noted in the ON group. (C) The serum concentrations of IP-10 in the NMOSD patients during both the acute and stable phases were higher than those seen in the healthy controls, as determined using the ELISA method. (D) The concentrations of IP-10 produced by the cells were significantly decreased in the remission phase in all eight NMOSD patients. (E) Correlation between the IP-10 protein levels in the cells after exposure to acute phase NMOSD sera and the albumin ratio (Q Alb). A higher concentration of IP-10 in the cells was significantly associated with a higher Q Alb value. Healthy control: cells exposed to sera from healthy controls; RRMS: cells exposed to sera from patients with RRMS; Acute NMO: cells exposed to sera from NMOSD patients during the acute phase; definite NMO: cells exposed to sera from definite NMOSD patients; LETM: cells exposed to sera from LETM patients; ON: cells exposed to sera from ON patients; Stable NMO: cells exposed to sera from NMOSD patients in the stable phase.

In addition, we examined the concentrations of IP-10 in equal amounts of protein (22.5 μl) obtained from the TY09 cells after exposure to the sera from the patients with NMO and RRMS as well as the healthy controls using another specified ELISA kit in order to confirm the reproducibility of the results obtained with the multiplexed fluorescent bead-based ELISA assay. Consequently, the level of IP-10 proteins produced by the cells in the acute NMOSD group were significantly increased compared to that observed in the RRMS, stable NMO and HC groups (**Fig. 2A**). We next compared the concentrations of IP-10 produced by the cells among the NMOSD subgroups, including the definite NMOSD, LETM and ON group. The IP-10 protein levels in the cells were significantly increased in all three subgroups of NMOSD compared to that seen in the RRMS and HC groups, and the level in the definite NMO group were significantly higher than those noted in the ON group (**Fig. 2B**). Furthermore, the analysis using sera obtained from the patients between the relapse and remission phases showed a significant decrease in the concentrations of IP-10 produced by the cells in the remission phase in all eight NMOSD patients (**Fig. 2D**). We also examined the concentrations of IP-10 in the serum samples obtained from the NMOSD patients, RRMS patients and healthy controls. As a result, the serum concentrations of IP-10 in the NMOSD patients during both the acute and stable phases were higher than those observed in the healthy controls, as determined using the ELISA method (**Fig. 2C**).

### Correlations between the clinical/laboratory/spinal MRI findings and the concentrations of IP-10 produced by cells after exposure to the NMOSD sera

We next determined the correlations between the clinical, laboratory and spinal MRI findings and the IP-10 concentrations in the cells after exposure to NMOSD. A higher concentration of IP-10 was correlated with a higher Q Alb level (**Fig. 2E**). In contrast, no significant differences were observed between the concentration of IP-10 and the disease duration, number of relapses, ΔEDSS, IgG index, lesion length or the appearance of Gd-enhanced lesions on MRI (**S1A–F Fig**).

## Discussion

A growing number of studies have focused on the important roles of cytokines and chemokines in the pathogenesis of NMO and have demonstrated that the levels of several cytokines/chemokines were elevated in the CSF of NMO patients during a relapse [13–17,25,26]. However, when the analyzing CSF sample from patients, it is difficult to discern which cytokines/chemokines are directly related to the pathogenesis, because some cytokines can be nonspecifically increased by CNS inflammation. To identify the pathogenic cytokines/chemokines that cause the disruption of the BBB in NMO, the present study examined the cytokine/chemokine profiles produced by BMECs as the result of cellular response after exposure to sera from patients with the active and stable phase of NMOSD, RRMS and healthy controls. The protein levels of IP-10, MCP-1, RANTES and IL-6 in the BMECs after they are challenged with sera from acute phase NMOSD patients were significantly increased compared to those from both RRMS patients and healthy controls. In addition, we also observed significantly higher levels of IP-10, MCP-1 and IL-6 after incubation of the cells with sera from acute phase NMOSD patients compared to stable phase NMOSD patients. Among these changed, a marked increase in the level of IP-10 produced by the cells after exposure to sera from the acute NMOSD patients was identified using multiplexed fluorescent bead-based ELISA assay. Up-regulation of IP-10 after incubation with the sera from three subtypes of acute NMOSD patients, including definite NMO, LETM and ON groups, was also observed with high reproducibility using another specified ELISA assay, and the sera from the definite NMOSD patients caused more severe BBB damage via IP-10 production compared to that induced by the sera from the ON patients. This effect was significantly decreased during the remission phase in the individual NMOSD patients. Moreover, the concentration of IP-10 produced by the cells significantly correlated with an increased Q Alb value, which may reflect BBB breakdown. Taken together, these findings suggest that the overproduction of IP-10 in BMECs induced by acute-phase NMOSD sera may be related to triggers of BBB breakdown in case of NMO.

Unexpectedly, our findings demonstrated that the serum concentrations of IP-10 was significantly higher in the NMOSD patients during both the acute and stable phases than in the healthy controls. We speculate that the serum concentrations of IP-10 in the acute phase of NMOSD patients are increased as a result of inflammation via the autocrine secretion of IP-10 by BMECs. We do not have a clear explanation as to why the increase in the IP-10 levels was observed in the even sera obtained during the stable phase in the NMOSD patients. However, we believe that this parameter may be increased nonspecifically, not in direct relation to the pathogenesis of NMOSD. The IP-10 levels in the CSF were significantly higher in NMO patients than in patients with other non-inflammatory neurological disorders, and correlated with the CSF cell counts and GFAP levels [17,27]. IP-10 can facilitate the trafficking of activated type 1 helper T cells and NK cells expressing the CXCR3 receptor across the BBB, and can regulate their recruitment to sites of inflammation [28]. A previous study demonstrated that treatment of an experimental autoimmune encephalomyelitis (EAE) with anti-IP-10 neutralizing antibodies reduced the accumulation of inflammatory mononuclear cells in the CNS and decreased the incidence of EAE development [29]. Our present results suggest that the humoral factors present in NMO sera during the acute phase markedly increase the production of IP-10 in BMECs via an autocrine mechanism, and can mediate their transfer of inflammatory cells to lesion sites. Our findings also indicated that measuring the CSF IP-10 level in NMO patients during the acute phase may be useful for monitoring the destruction of the BBB. In addition, together with the increase in IP-10 secretion by BMECs following exposure to NMO sera, increased levels of MCP-1 and RANTES may also be involved in the leukocyte transendothelial migration across the BBB during the acute phase of NMO, although previous reports have demonstrated that the levels of these markers were not elevated in the CSF samples from NMO patients [14]. Together, these results suggest that the humoral factors present in the sera from acute phase NMO patients, distinct from those present in MS patients, can increase the chemokine expression in the BBB, thereby inducing the migration of activated leukocytes in the CNS parenchyma.

In several studies, an elevation of the IL-6 level was observed in both CSF and serum samples from NMO patients, and this showed the strongest association with the clinical variables in these patients [15–17]. A previous study by another group and a recent study by our group suggested that the CSF IL-6 is mainly produced by astrocytes in NMOSD [30,31]. However, our present study demonstrated that the release of IL-6 from BMECs is significantly higher in the acute phase NMO patients than in MS patients, healthy controls and even stable phase NMO patients, thus suggesting that BMECs, as well as astrocytes, may be a major source of IL-6 production during the acute phase of NMO. Tocilizumab is a novel humanized monoclonal antibody against IL-6, and may be effective as a maintenance treatment for NMO patients who fail to achieve sustained remission after conventional therapy [32,33]. It remains unclear whether this antibody can penetrate the BBB and affect astrocytes under normal conditions. We speculate that the intravenous administration of this antibody may act primarily on BMECs by blocking the IL-6 receptor expressed on their surface, thereby preventing the disruption of the BBB, and resulting in a decrease in the pathogenesis of NMO.

We also observed significant downregulation of the IL-1β, IL-10 and TNF-α produced by the BMECs following exposure to sera from the acute phase NMOSD patients compared to the samples from patients with the stable phase NMOSD, MS and healthy controls. Several studies have shown that both IL-1β and TNF-α are important proinflammatory cytokines that are responsible for disrupting the BBB [34,35], while IL-10 is considered to be an anti-inflammatory cytokine capable of inhibiting proinflammatory responses [36], although the levels of these three cytokine were all reported to be elevated in the CSF samples from NMOSD patients. It is possible that these three cytokines are massively secreted by BMECs following exposure to the acute phase NMO sera and are eventually exhausted; however, the concentrations of these cytokines in the culture supernatant did not show any significant difference between the groups in our study (data not shown). Both the increase in the PDGF-BB protein and the decrease in the IL-4 protein produced by BMECs are considered to be non-specific for acute NMOSD, because these changes were observed in both the acute NMOSD group and in the MS group.

Our previous study focused on the contributions of autocrine VEGF produced in response to exposure to sera to the disruption of the BBB in both NMO and RRMS patients [20,37]. In the current study, the protein levels of VEGF produced by the BMECs were significantly increased following incubation with sera from the RRMS patients and stable phase NMOSD patients compared to those following exposure to sera from healthy controls. The results showing that the production of VEGF by BMECs did not significantly change after exposure to sera from the acute phase NMO patients does not conflict with our previous study [20], in which the secretion of VEGF in BMECs was upregulated following exposure to sera from anti-BMECs antibody-positive NMO patients, but not from anti-BMECs antibody-negative NMO patients, so that those did not change as a mass by exposure to sera including both NMO patients with and without anti-BMECs antibodies.

In conclusion, our study showed that there was marked elevation of the IP-10 levels produced by the BBB (as represented by BMECs) induced by humoral factors present in the sera during the acute phase of NMO. Our findings shed further light on the importance of IP-10 production by the BBB as a novel biomarker to evaluate the BBB disruption in NMO patients, and may also represent a promising new target for the treatment of the disease. A further understanding of the pathogenic cytokines and chemokines responsible for the breakdown of the BBB in NMO could facilitate the development of novel therapies.

## Supporting Information

S1 Fig Correlation between the concentrations of IP-10 in the cells after exposure to NMOSD sera and the clinical/laboratory/spinal MRI findings. There were no significant differences between the concentrations of IP-10 in the cells after exposure to the acute-phase NMOSD sera and the disease duration (A), number of relapses (B), ΔEDSS (C), IgG index (D), length of spinal lesions on MRI (E) or presence of Gd-enhanced lesions on MRI (F).
(TIF)

S1 Dataset Raw data of multiplexed fluorescent bead-based immunoassay regarding the concentration of cytokine/chemokine secreted by BMECs after exposure to patient’s sera Abbreviations: HC = healthy control; RRMS = relapse-remiting multiple sclerosis; definite NMO = definite neuromyelitis optica in the acute phase; ON = isolated optic neuritis in the acute phase; LETM = isolated longitudinally extensive transverse myelitis in the acute phase; stable NMO = neuromyelitis optica in the stable phase; IP = induced protein; IL = interleukin; MCP = monocyte chemotactic protein; RANTES = regulated upon activation, normal T cell expressed and secreted; PDGF = platelet-derived growth factor; VEGF = vascular endothelial growth factor; TNF = tumor necrosis factor; IL-1ra = interleukin-1 receptor antagonist; FGF = fibroblast growth factor; G-CSF = granulocyte colony-stimulating factor; MIP = macrophage inflammatory protein; GM-CSF = granulocyte-macrophage colony-stimulating factor Unit: pg/ml (XLSX)
