# Supplementary figures and images for: Markedly Increased IP-10 Production by Blood-Brain Barrier in Neuromyelitis Optica
Source: PLoS One. 2015 Mar 26;10(3):e0122000. doi: 10.1371/journal.pone.0122000 (PMC4374747; doi:10.1371/journal.pone.0122000)

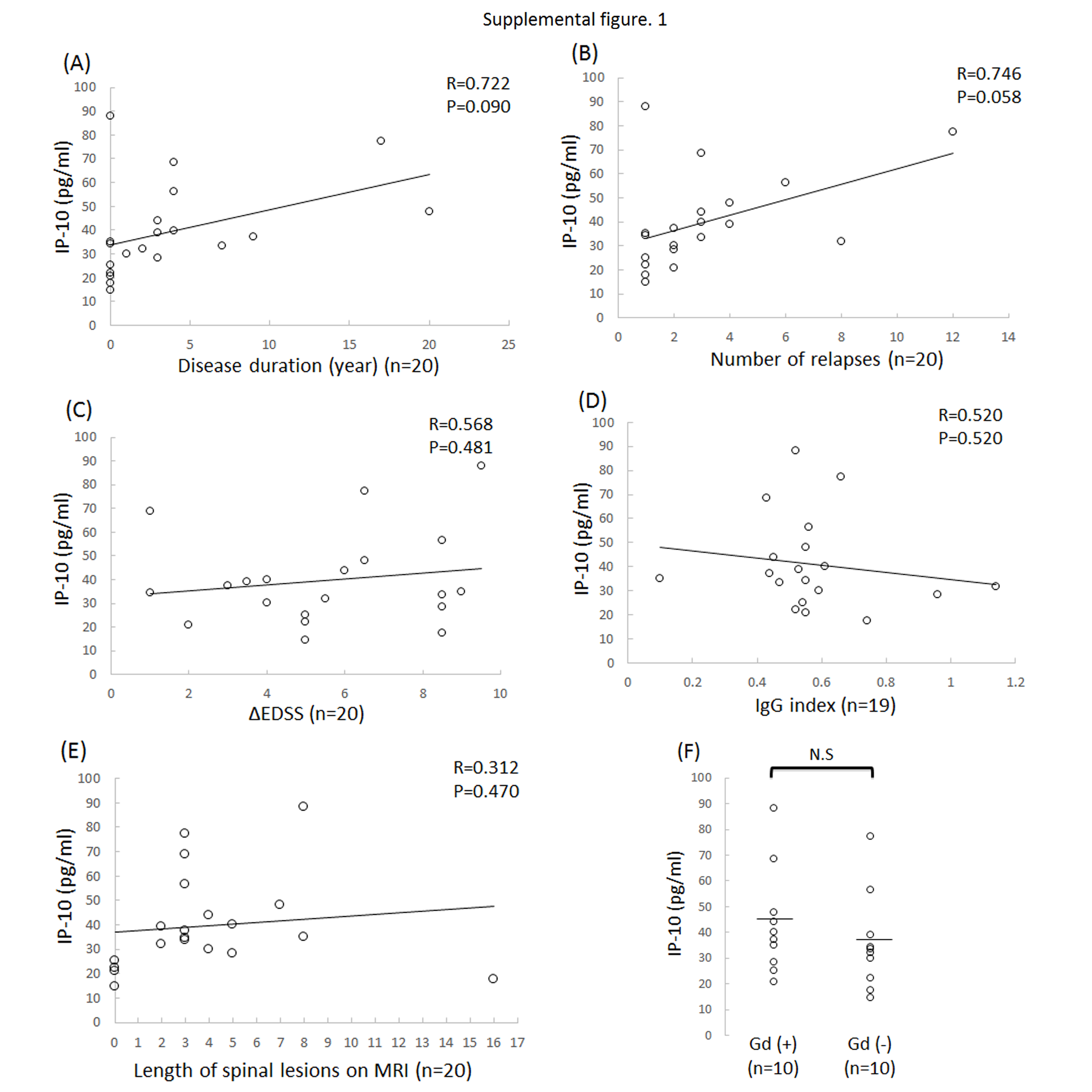

Supplement: S1 Fig — There were no significant differences between the concentrations of IP-10 in the cells after exposure to the acute-phase NMOSD sera and the disease duration (A), number of relapses (B), ΔEDSS (C), IgG index (D), length of spinal lesions on MRI (E) or presence of Gd-enhanced lesions on MRI (F). (TIF) [file pone.0122000.s001.tif]
